# Umbilical Cord-Derived Mesenchymal Stem Cells Suppress Autophagy of T Cells in Patients with Systemic Lupus Erythematosus via Transfer of Mitochondria

**DOI:** 10.1155/2016/4062789

**Published:** 2016-12-07

**Authors:** Jinyun Chen, Qian Wang, Xuebing Feng, Zhuoya Zhang, Linyu Geng, Ting Xu, Dandan Wang, Lingyun Sun

**Affiliations:** ^1^Department of Rheumatology and Immunology, The Affiliated Drum Tower Hospital of Nanjing University Medical School, Nanjing, Jiangsu 210008, China; ^2^Department of Rheumatology and Immunology, Drum Tower Clinical Medical College of Nanjing Medical University, Nanjing, Jiangsu 210008, China; ^3^Department of Rheumatology and Immunology, The 3rd Affiliated Hospital of Soochow University, Suzhou, Jiangsu 215006, China

## Abstract

Aberrant autophagy played an important role in the pathogenesis of autoimmune diseases, especially in systemic lupus erythematosus (SLE). In this study, we showed that T cells from SLE patients had higher autophagic activity than that from healthy controls. A correlation between autophagic activity and apoptotic rate was observed in activated T cells. Moreover, activation of autophagy with rapamycin increased T cell apoptosis, whereas inhibition of autophagy with 3-MA decreased T cell apoptosis. Umbilical cord-derived mesenchymal stem cells (UC-MSCs) could inhibit respiratory mitochondrial biogenesis in activated T cells to downregulate autophagy and consequently decrease T cell apoptosis through mitochondrial transfer and thus may play an important role in SLE treatment.

## 1. Introduction

Mesenchymal stem cells, or MSCs, are multipotent stromal cells that can differentiate into a variety of cell types, including osteoblasts, chondrocytes, myocytes, and adipocytes [1]. They can be derived from bone marrow and other nonmarrow tissues, such as the placenta, umbilical cord blood, adipose tissue, adult muscle, corneal stroma, or the dental pulp of deciduous baby teeth. Besides differentiation capacity, numerous studies have demonstrated MSCs have immunomodulatory effects [2]. And transplantation of umbilical cord-derived mesenchymal stem cells (UC-MSCs) has been shown effective in patients with autoimmune diseases, especially systemic lupus erythematosus (SLE) [3–5]. UC-MSCs could inhibit T cell proliferation in lupus patients by secreting large amounts of indoleamine 2,3-dioxygenase (IDO), which is enhanced by IFN-*γ* produced by CD8+T cells [6]. They also suppress B cell proliferation and differentiation [7] and regulate Treg/Th17 balance in SLE patients [8]. However, their regulation on autophagy has not been assessed. Recent works indicated the important role of autophagy in SLE [9, 10], which demonstrated that T cells from SLE patients have overactivated autophagy [11, 12].

Autophagy is the natural, destructive mechanism that disassembles, through a regulated process, unnecessary or dysfunctional cellular components. Peripheral T cells from SLE patients have been shown to have defective mitochondria, demonstrated by mitochondrial hyperpolarization and ATP depletion [13]. Prolonged autophagy activation usually leads to a high turnover rate of proteins and organelles, and a high rate above the survival threshold could result in cell death [14] and thus may be related to increased apoptosis of peripheral T cells from SLE patients [15]. In this study, the link between autophagy and apoptosis of activated T cells from SLE patients was addressed and the regulation of UC-MSCs on T cell autophagy was investigated.

## 2. Materials and Methods

### 2.1. Patients and Healthy Controls

We included 32 SLE patients (male : female = 7 : 25, mean age = 36, range 16 to 49 years old) who were diagnosed according to the classification criteria of the American College of Rheumatology [16]. Current SLE disease activity was measured using the SLE Disease Activity Index (SLEDAI) [17]. The mean ± standard error of mean (SEM) SLEDAI score was 6.5 ± 0.9. Among these patients, 9 were measured for basal autophagic activity and mitochondrial mass (detailed clinical characteristics and laboratory features were shown in Table 1). 30 healthy donors (male : female = 8 : 22, mean age = 33, range 22 to 51 years old) were recruited as controls. They were recruited from the Affiliated Drum Tower Hospital of Nanjing University Medical School after informed consent was obtained. The protocol was approved by the Ethics Committee at the Affiliated Drum Tower Hospital of Nanjing University Medical School.

### 2.2. UC-MSCs Isolation and Culture

Umbilical cords (UC) were obtained from local maternity hospitals after normal deliveries and then digested and cultured for 2 generations. After 2 passages, UC-MSCs were harvested. Flow cytometry analyses showed CD29, CD44, and CD105 expression >95%, in parallel with CD45, CD34, CD14, and HLA-DR expression <2%. All antibodies that used for flow cytometry analyses were purchased from eBioscience.

### 2.3. Western Blotting

CD3+T cells were purified from PBMCs by microbeads and lysed with SDS sample buffer containing 20 mM Tris-HCl (pH 7.6), 250 mM NaCl, 0.5% NP-40, 3 mM ethylenediaminetetraacetic acid, and 1.5 mM ethyleneglycoltetraacetic acid with 10 mg/mL aprotinin, 10 mg/mL leupeptin, 1 mM DTT, 1 mM paranitrophenylphosphate, and 0.1 mM Na3VO4 as protease and phosphatase inhibitor. Cell lysates were separated by sodium dodecyl sulfate-polyacrylamide gel electrophoresis (SDS-PAGE) and transferred to a polyvinylidene difluoride (PVDF) membrane (Millipore). Blots were probed by anti-LC3B antibody (Cell Signaling Technology, Inc.), anti-p62 antibodies (Cell Signaling Technology, Inc.), and anti-GAPDH antibody (Cell Signaling Technology, Inc.) before visualizing with horseradish peroxidaseconjugated secondary antibodies followed by development with FluorChem FC2 System (Alpha Innotech Corporation).

### 2.4. Flow Cytometry

For detection of apoptosis, T cells were stained for surface marker using the following antibodies: CD3− PE (eBioscience, 12-0037-42), CD4− APC (eBioscience, 17-0049-42), CD8− PercP-Cy5.5 (eBioscience, 45-0088-42), and Annexin V-FITC (BD Pharmingen, 559763). Apoptosis was defined as Annexin V (+).

Intracellular staining for membrane-attached LC3II on T cells was performed according to the manufacturer's instructions (FlowCellect Autophagy LC3 antibody based kit, Merck&Millipore, FCCH100171). And autophagy was measured using LC3-FITC MFI (Mean Fluorescence Intensity).

Apoptosis was measured after autophagy activation with rapamycin (Cayman, 53123-88-9) or AMPK Activator VI RSVA314 (Merck&Millipore, 171272-10MGCN) and after being treated with autophagy suppressor 3-MA (Cayman, 13242-50 mg) or chloroquine (Sigma-Aldrich, C6628-25G).

Respiratory mitochondrial mass (mitochondria that maintains transmembrane potential) was detected with MitoTracker Deep Red (Invitrogen, M22426), and total mitochondrial mass (including respiratory mitochondria and mitochondria that have lost transmembrane potential) was detected with MitoTracker Green (Invitrogen, M7514) staining. Data were collected on FACS Calibur (BD Bioscience) and analyzed using FlowJo software (Tree Star).

### 2.5. Fluorescence Microscopy

PBMCs were labeled with CFSE (eBioscience, 65-0850-84) after isolation and stimulated with anti-CD3/CD28 for 16 h. UC-MSCs were labeled with MitoTracker Deep Red. After coculturing for 6 h, PBMCs were filmed with a fluorescence microscope (Olympus FSX100).

### 2.6. RNA Interference Transfection in Human T Lymphocytes

The silencing of ATG5 was performed with a chemically synthesized siRNA (5′-AACCUUUGGCCUAAGAAGAdTdT-3′; Bioinshine, China) and a control siRNA (Bioinshine, China) with a random sequence not present in the human genome (5′-UUCUCCGAACGUGUCACGUdTdT-3′). 10^6^ T lymphocytes were resuspended in 250 *μ*l of siRNA-lipid complex (Lipofectamine RNAiMAX Reagent, Invitrogen) and then incubated in complete medium for 48 h before harvesting. Apoptosis of T lymphocytes was detected by flow cytometry.

### 2.7. Statistical Analysis

Data were summarized as mean ± SEM. Statistical significance was performed by Student's *t*-test and the correlation coefficient between autophagy and apoptosis was analyzed by linear regression. A *p* value < 0.05 was considered statistically significant. In order to avoid the influence of different cell counts among data, some flow cytometry results were shown as “normalized to mode” (mode mean “the most frequent value in a data set”) which was a ratio of cell numbers. All statistical analyses were performed using GraphPad Prism software (Graph-Pad, San Diego, CA, USA) or SPSS version 13.0 statistical software (IBM SPSS, Chicago, IL, USA).

## 3. Results

### 3.1. Autophagy Increased in T Cells from SLE Patients

**Figure 1:**
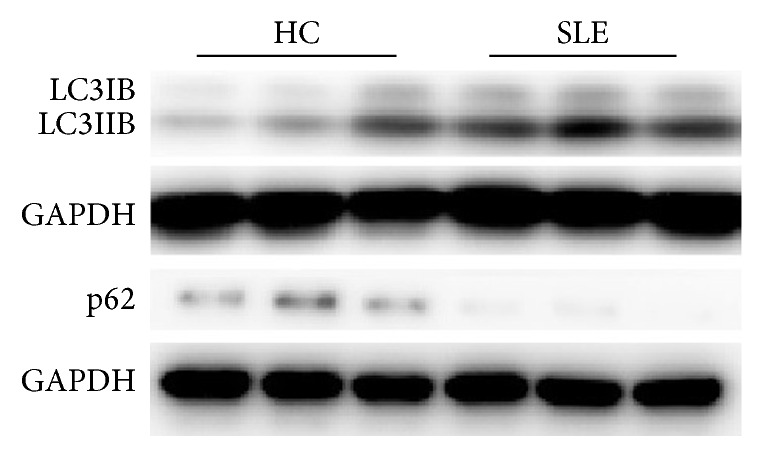
T cells from SLE patients had overactivated autophagy. CD3+T cells were purified from peripheral blood mononuclear cells (PBMCs). T cells from SLE patients had higher expression of LC3IIB and lower p62 than that from healthy controls (HC).

**Figure 2:**
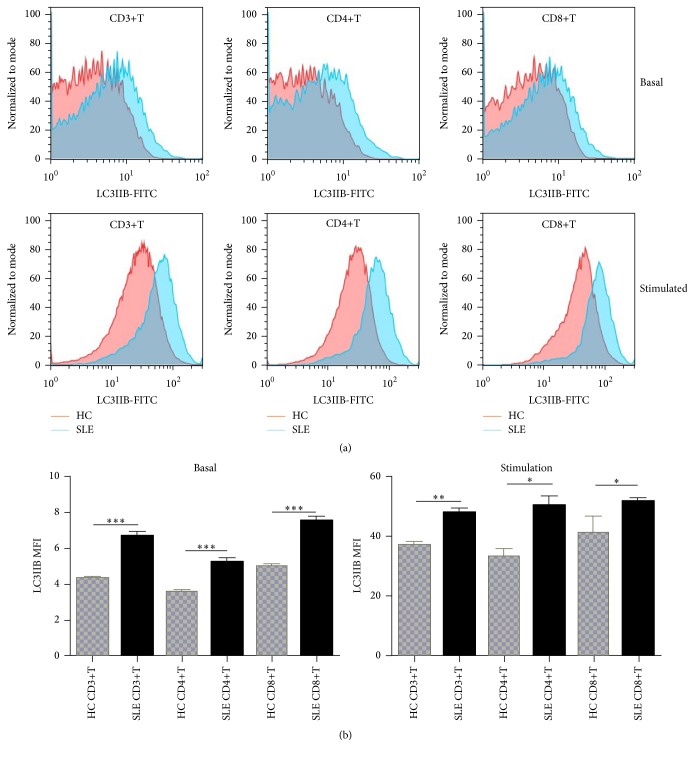
T cells from SLE patients had overactivated autophagy. PBMCs were stimulated with or without anti-CD3/CD28 antibodies (3 *μ*g/mL) for 3 days. In vitro experiments were performed in triplicate. ((a), (b)) Basal autophagy of T cells from SLE patients (*n* = 9) was higher than that from healthy controls (*n* = 5) and further increased after anti-CD3/28 stimulation. The number of T cells was normalized to mode. ^*∗*^ *p* < 0.05;  ^*∗∗*^ *p* < 0.01;  ^*∗∗∗*^ *p* < 0.001.

To evaluate the autophagic activity of T cells, we detected the level of autophagic LC3-IIB by western blot and flow cytometry. Basal autophagic activity of peripheral T cells was detected immediately after isolation of PBMCs. Expression of LC3IIB was markedly higher and p62 lower in CD3+T cells from SLE patients (Figure 1). Further detection of LC3IIB by flow cytometry also indicated higher basal autophagic activity in SLE patients in comparison to healthy controls (6.69 ± 0.23 versus 4.31 ± 0.13, *p* < 0.0001 for CD3+T cells; 5.25 ± 0.22 versus 3.58 ± 0.07, *p* = 0.0001 for CD4+T cells; 7.52 ± 0.26 versus 5.01 ± 0.09, *p* < 0.0001 for CD8+T cells) (Figure 2). And autophagy of CD4+T cells was significantly correlated with SLEDAI score (*p* = 0.014, *r* = 0.815). Additionally, T cells from SLE patients had higher autophagic activity than that from controls after anti-CD3/CD28 stimulation (48.07 ± 1.51 versus 37.00 ± 1.00, *p* = 0.0077 for CD3+T cells; 50.38 ± 3.02 versus 33.20 ± 2.30, *p* = 0.0213 for CD4+T cells; 51.64 ± 1.10 versus 41.20 ± 5.20, *p* = 0.0254 for CD8+T cells) (Figure 2).

### 3.2. T Cells with Increased Autophagy Were Prone to Apoptosis

**Figure 3:**
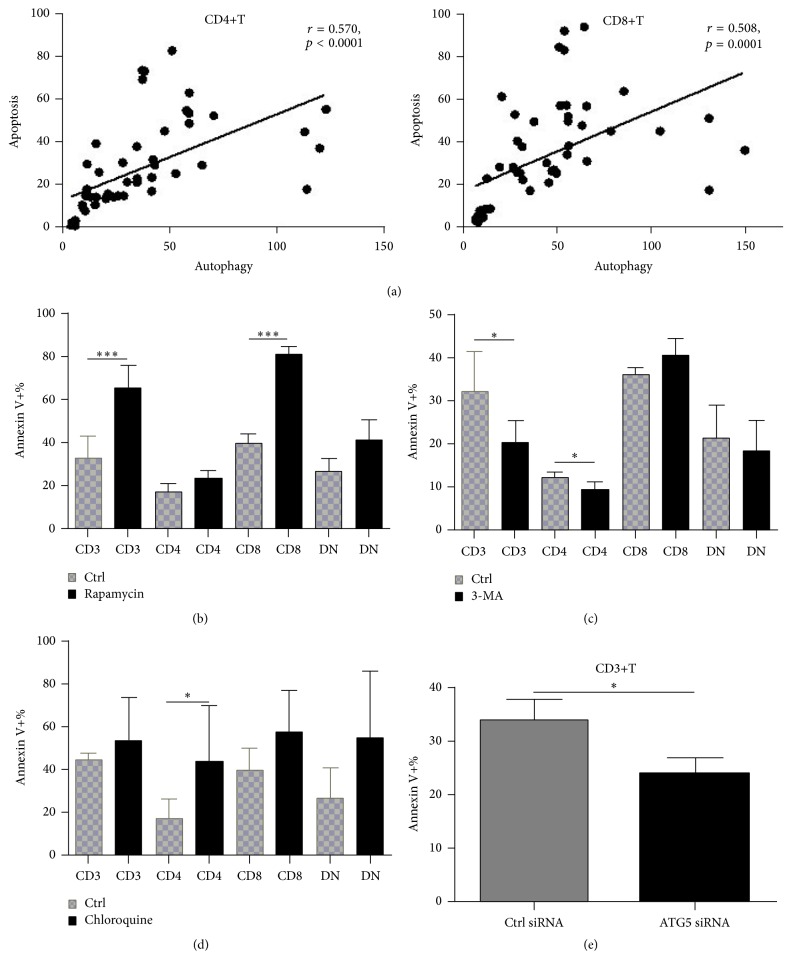
T cells with increased autophagy following stimulation were prone to apoptosis. Autophagy and apoptosis of T cells from SLE patients were detected after stimulation with anti-CD3/C28 antibodies for different time. And their correlation was analyzed. Apoptosis was also measured after autophagy activation with rapamycin (50 ng/mL, 72 h, simultaneously with anti-CD3/28 stimulation), or suppression with 3-methyladenine (3-MA) (5 mM, 6 h, following anti-CD3/28 stimulation for 48 h) or chloroquine (CQ, 50 *μ*M for 24 h, following anti-CD3/28 stimulation for 48 h). In vitro experiments were performed in triplicate. (a) Correlation of apoptosis of both CD4+T and CD8+T cells from SLE patients with autophagy level following anti-CD3/28 stimulation (*n* = 32). (b) Activation of autophagy with rapamycin further promote apoptosis of T cells, especially in CD8+T cells (*n* = 7). (c) Suppression of autophagy with 3-MA rescued T cells (mainly CD4+T) from apoptosis (*n* = 7). (d) Treatment with chloroquine increased apoptosis of CD4+T cells (*n* = 6). (e) ATG5 silencing decrease apoptosis of T cells significantly.  ^*∗*^ *p* < 0.05;  ^*∗∗∗*^ *p* < 0.001.

Besides elevated autophagy, apoptosis of T cells from SLE patients increased following stimulation and it was positively associated with autophagy (*r* = 0.570, *p* < 0.0001 for CD4+T cells; *r* = 0.508, *p* = 0.0001 for CD8+T cells) (Figure 3(a)). Meanwhile, activation of autophagy with rapamycin further increased T cell apoptosis (65.31% ± 10.57% versus 33.07% ± 10.03%,  *p* = 0.0001), especially in CD8+T cells (80.80% ± 3.60% versus 39.72% ± 4.40%,  *p* < 0.0001) (Figure 3(b)). However, inhibition of autophagy with 3-MA decreased T cell apoptosis (20.37% ± 5.15% versus 32.13% ± 9.37%,  *p* = 0.0361), mainly in CD4+T cells (9.45% ± 1.72% versus 12.05% ± 1.50%,  *p* = 0.0469) (Figure 3(c)), further confirming the relationship between autophagy and apoptosis of T cells from SLE patients. However, treatment with chloroquine (CQ) further increased apoptosis of CD4+T cells (43.90% ± 11.70% versus 17.33% ± 3.59%,  *p* = 0.0429), maybe due to lysosomal degradation blockade (Figure 3(d)). We also observed a significant decrease of apoptosis of CD3+T cells after ATG5 silencing with siRNA (Figure 3(e)). These results suggested that T cells from SLE patients were prone to apoptosis after autophagy activation.

### 3.3. SLE Patients Had Excessive Mitochondria within T Cells

**Figure 4:**
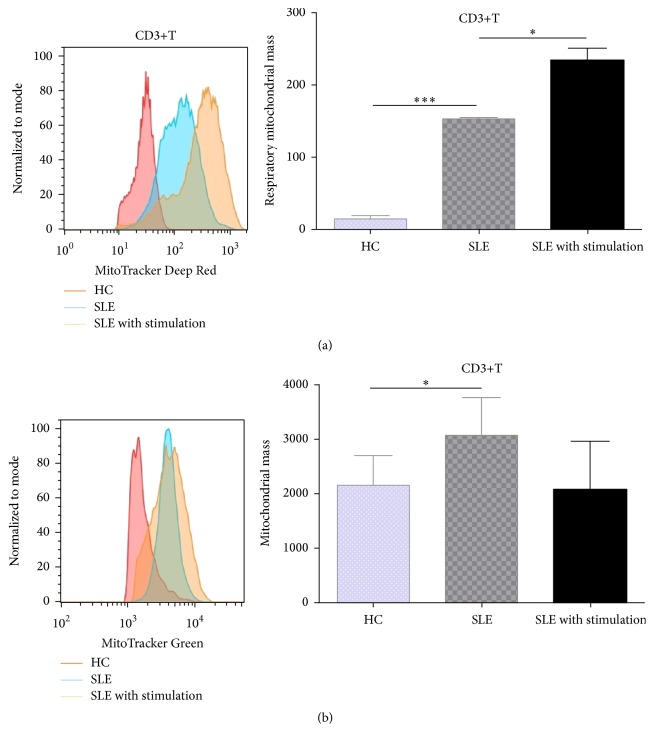
SLE patients had excessive mitochondria within T cells. PBMCs were stimulated with or without anti-CD3/CD28 antibodies (3 *μ*g/mL) for 3 days. In vitro experiments were performed in triplicate. Both respiratory (a) and total mitochondrial mass (b) of CD3+T cells from systemic lupus erythematosus (SLE) patients (*n* = 9) were significantly higher than that from healthy controls (*n* = 5), and respiratory mitochondrial mass further increased following anti-CD3/CD28 activation. ^*∗*^ *p* < 0.05, ^*∗∗∗*^ *p* < 0.001.

As autophagy was essential for mitochondrial turnover, we detected mitochondrial mass in T cells from SLE patients. With the use of MitoTracker Deep Red (MDR) and MitoTracker Green (MG) staining, we found T cells from SLE patients had elevated respiratory mitochondrial mass (152.50 ± 1.50 versus 13.52 ± 3.54, *p* < 0.0001) and total mitochondrial mass (3079.0 ± 348.5 versus 2165.0 ± 218.1, *p* = 0.0461) in comparison to that from controls (Figure 4). Respiratory mitochondrial mass of T cells from SLE patients further increased following anti-CD3/28 stimulation (232.70 ± 1.5017.80 versus 152.50 ± 1.50,  *p* = 0.0462) (Figure 4(a)). As we know, cell activation would induce increased cellular biogenesis which is ATP-dependent, and mitochondria are the main source of ATP synthesis. However, some studies showed that the mitochondrial function of lymphocytes from SLE patients was defective, characterized by high mitochondrial transmembrane potential (ΔΨm) and low ATP production [13, 18]. These results indicated that T cell activation may increase mitochondrial biogenesis in SLE patients, but it did not improve their energy “starvation” state and activated autophagy failed to eliminate the excessive mitochondria in those T cells. As T cells from SLE patients were characterized by increased ΔΨm, we detected the defective mitochondria in them with potential-dependent probe, MDR, in the following experiments.

### 3.4. UC-MSCs Decreased Respiratory Mitochondrial Accumulation and Suppressed Autophagy of T Cells

**Figure 5:**
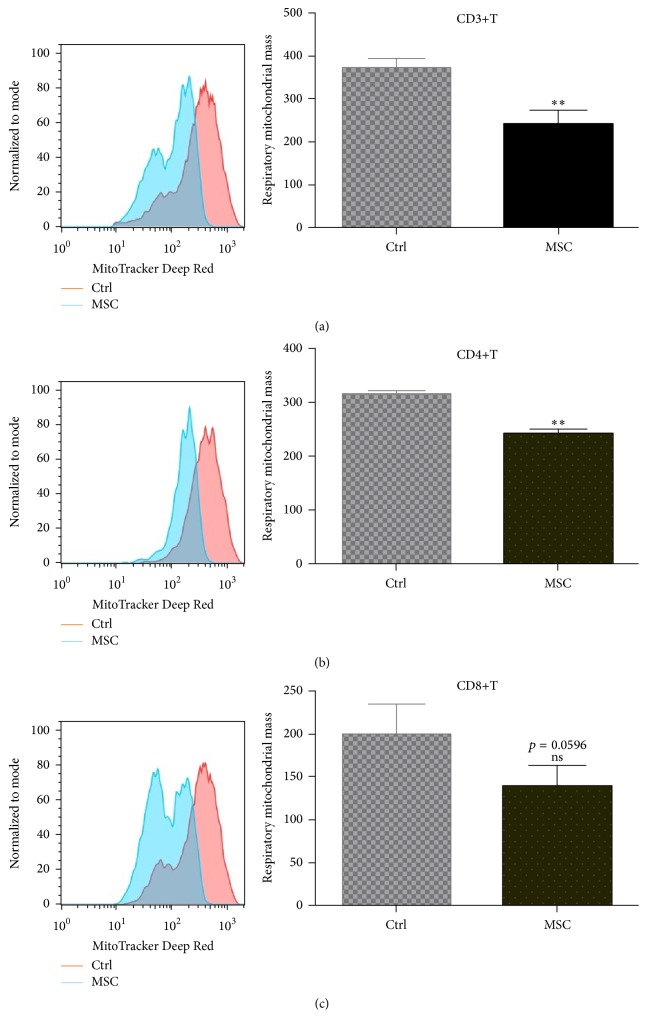
UC-MSCs inhibited respiratory mitochondrial accumulation in T cells from SLE patients. PBMCs from SLE patients were activated with anti-CD3/CD28 antibodies and cultured with or without mesenchymal stem cells (UC-MSCs) for 72 h. All experiments were performed in triplicate. (a–c) UC-MSCs inhibited respiratory mitochondrial accumulation in activated CD3+T, CD4+T, and CD8+T cells (*n* = 4). ns, not significant;  ^*∗∗*^ *p* < 0.01.

**Figure 6:**
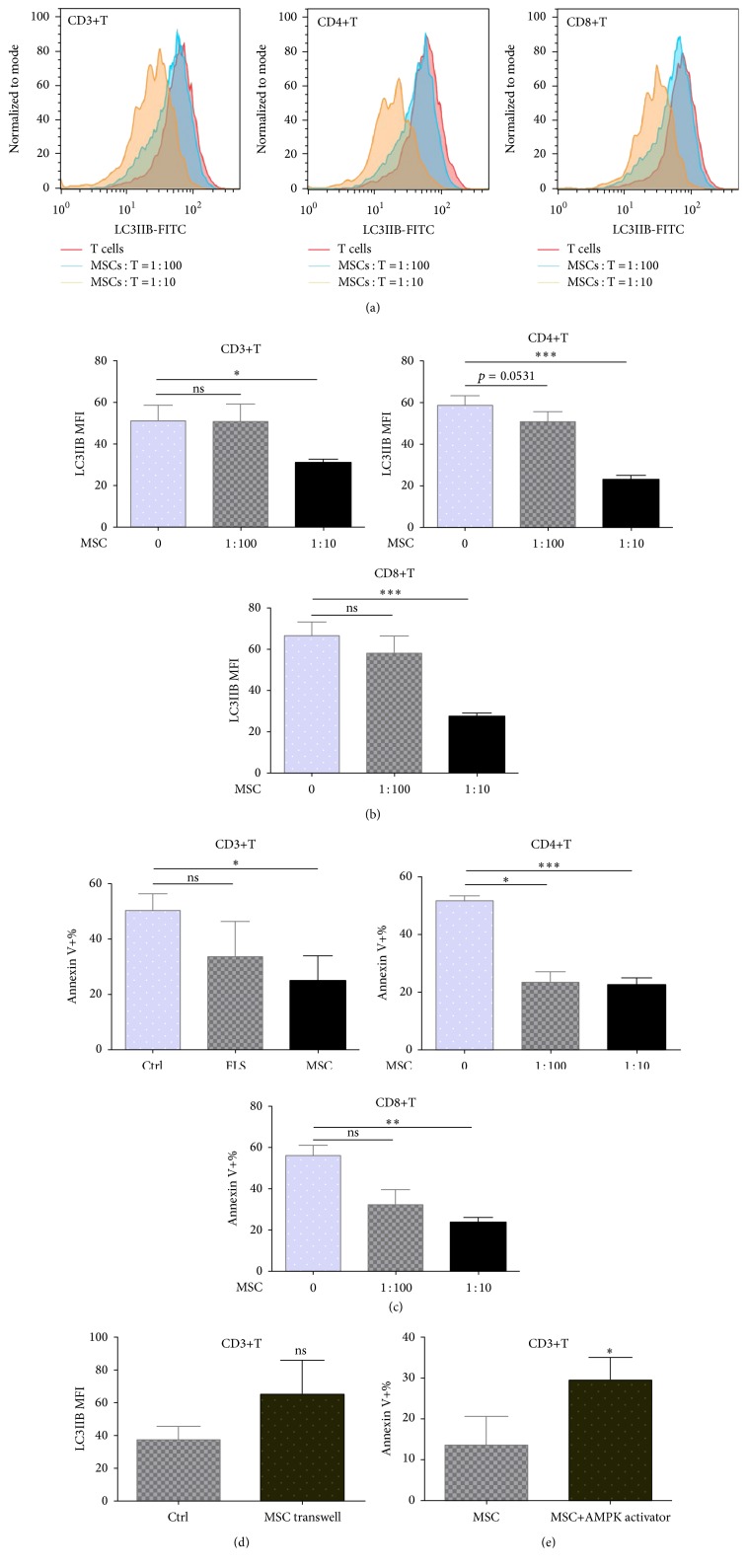
UC-MSCs rescued T cells from apoptosis by inhibiting autophagy activation of T cells in cell contact-dependent way. PBMCs from SLE patients were cultured with or without UC-MSCs (UC-MSCs : T = 1 : 100 or 1 : 10) and stimulated with anti-CD3/CD28 antibodies (3 *μ*g/mL) for 3 days. All experiments were performed in triplicate. (a) Representatives of UC-MSCs' inhibition on autophagy of CD3+T cells, CD4+T cells, and CD8+T cells, respectively. (b) UC-MSCs inhibited autophagy of CD3+T, CD4+T, and CD8+T cells dose-dependently (*n* = 7). (c) UC-MSCs suppressed T cell apoptosis in a dose-dependent way (*n* = 7). (d) Coculture with UC-MSCs via transwell could not inhibit T cell autophagy (*n* = 3). (e) Treatment of T cells with AMP-activated protein kinase (AMPK) activator abrogated UC-MSCs' inhibition on T cell apoptosis (*n* = 3). ns, not significant;  ^*∗*^ *p* < 0.05;  ^*∗∗*^ *p* < 0.01;  ^*∗∗∗*^ *p* < 0.001.

To investigate whether UC-MSCs could regulate T cell autophagy, PBMCs from SLE patients were cultured with or without UC-MSCs (T : UC-MSCs = 10 : 1) in vitro for 3 days (with anti-CD3/CD28 stimulation). As shown in Figure 5, respiratory mitochondrial mass of T cells decreased markedly after cocultured with UC-MSCs (241.00 ± 32.86 versus 371.30 ± 22.03, *p* = 0.002 for CD3+T cells; 242.50 ± 8.38 versus 315.8 ± 5.45,  *p* = 0.0034 for CD4+T cells; 139.80 ± 23.5 versus 199.00 ± 35.74, *p* = 0.0596 for CD8+T cells). Subsequently, T cell autophagy (30.70 ± 1.76 versus 51.37 ± 7.07, *p* = 0.0469 for CD3+T cells; 22.47 ± 2.41 versus 58.78 ± 4.68,  *p* < 0.0001 for CD4+T cells; 27.16 ± 1.87 versus 67.00 ± 6.32,  *p* < 0.0001 for CD8+T cells) and apoptosis (24.31% ± 9.47% versus 50.10% ± 6.33%,  *p* = 0.0432 for CD3+T cells; 22.20% ± 2.60% versus 51.93% ± 1.77%,  *p* = 0.0003 for CD4+T cells; 23.25% ± 2.43% versus 55.87% ± 4.63%,  *p* = 0.0011 for CD8+T cells) significantly decreased as well (Figures 6(a)–6(c)), which was dose (Figures 6(b) and 6(c)) and cell contact dependent (Figure 6(d)). However, AMPK activation of T cells with RSVA314 abrogated UC-MSCs' inhibition on T cell apoptosis (29.50% ± 5.58% versus 13.57% ± 7.31%, *p* = 0.0266 for CD3+T cells) (Figure 6(e)), suggesting that UC-MSCs rescued T cells from apoptosis via improving their energy “starvation.”

### 3.5. UC-MSCs Transferred Functional Mitochondria to Activate T Cells

**Figure 7:**
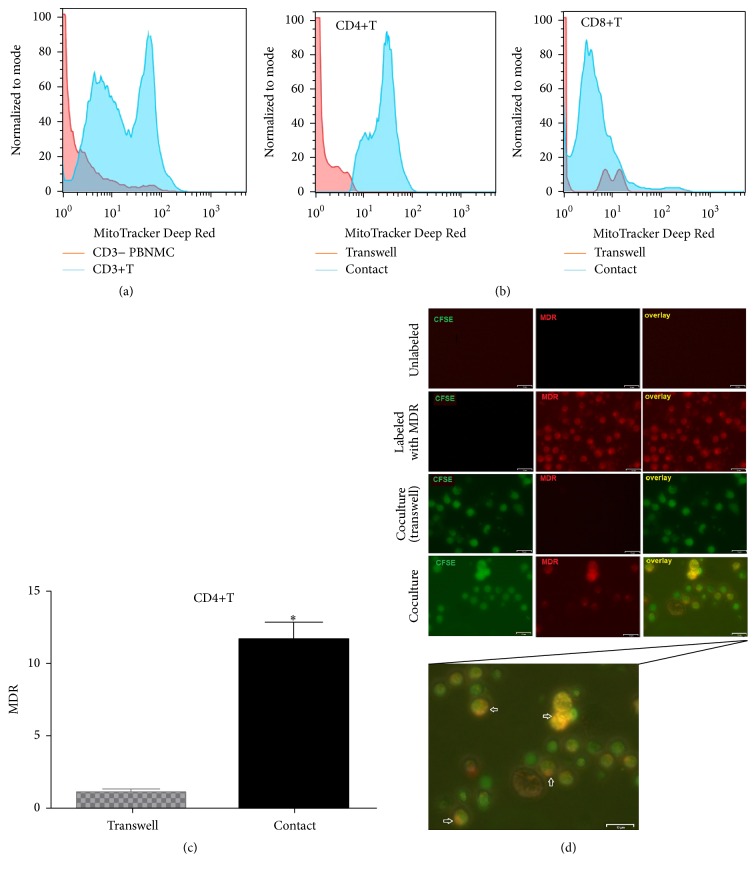
UC-MSCs transferred mitochondria to activate T cells. PBMCs from SLE patients were labeled with carboxyfluorescein succinimidyl amino ester (CFSE) and treated with anti-CD3/CD28 antibodies for two days. Then they were cocultured with UC-MSCs for 12 h, which had been prelabeled with respiratory mitochondrion specific probe Mitotracker Deep Red (MDR). PBMCs were cultured with UC-MSCs through transwell as control. Then PBMCs were stained with anti-CD3, anti-CD4, or anti-CD8 dye and detected for MDR fluorescence with flow cytometry. Fluorescence microscopy was carried out similarly with anti-CD3/28 stimulation for 12 h and then cocultured for 6 h. All experiments were performed in triplicate. (a) T cells (CD3 positive) rather than non-T cells (CD3 negative) got MDR staining. (b–d) T cells cultured with UC-MSCs directly rather than through transwell got transferred mitochondria (*n* = 3). Arrows indicated transferred mitochondria within lymphocytes.  ^*∗*^ *p* < 0.05.

The above results demonstrated that UC-MSCs definitely could inhibit respiratory mitochondrial accumulation and autophagy activation of T cells from SLE patients. Furthermore, we found UC-MSCs transferred functional mitochondria to T cells to improve their energy “starvation.” In these experiments, UC-MSCs were prestained with MDR and then cultured with PBMCs which had been stimulated with anti-CD3/CD28 antibodies. As demonstrated, activated CD3+T cells got much more stained mitochondria than other cells (CD3 negative) in PBMCs (Figure 7(a)) and the effect was cell-to-cell contact-dependent (11.61 ± 1.34 versus 1.23 ± 0.11, *p* = 0.0136 for CD4+T cells) (Figures 7(b) and 7(c)). Further studies using fluorescence microscopy confirmed that UC-MSCs transferred their functional mitochondria to T cells (Figure 7(d)). Interestingly, we could distinguish two populations of CD3+T cells (Figure 7(a)), and CD4+T cells got more mitochondria than CD8+T cells (Figure 7(b)), indicating there was a precedence of mitochondrial transfer for CD4+T cells. In summary, UC-MSCs may improve energy state of T cells via mitochondrial transfer and thus suppressed excessive mitochondrial biogenesis and accumulation, which finally rescued them from activated autophagy and apoptosis.

### 3.6. Functional Mitochondria Were Essential for UC-MSCs to Regulate T Cell Autophagy

**Figure 8:**
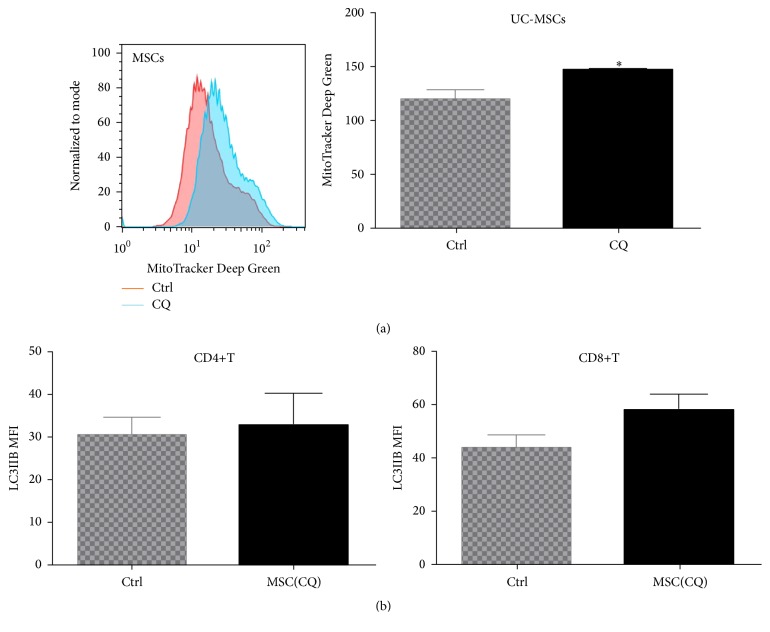
Functional mitochondria were essential for UC-MSCs to regulate T cell autophagy. UC-MSCs labeled with MG were treated with chloroquine (CQ, 50 *μ*M for 24 h) to block mitochondrial turnover. Then PBMCs were stimulated with anti-CD3/28 and cultured with CQ-treated UC-MSCs for 3 days. All experiments were performed in triplicate. (a) CQ treatment caused mitochondrial accumulation in UC-MSCs (*n* = 4). (b) UC-MSCs with accumulated mitochondria lost the ability to regulate T cell autophagy (*n* = 4).  ^*∗*^ *p* < 0.05.

To confirm the essential role of mitochondria in autophagy regulation, we used chloroquine, an autophagy-lysosomal inhibitor, to block late-stage autophagy of UC-MSCs. As expected, we found blockade of autophagic flux caused accumulation of mitochondria in UC-MSCs, indicating impairment to eliminate excessive or dysfunctional mitochondria (145.0 ± 2.65 versus 119.50 ± 8.50, *p* = 0.0383) (Figure 8(a)). Interestingly, it subsequently abrogated UC-MSCs' regulation on T cell autophagy (Figure 8(b)), which suggested that UC-MSCs could not regulate T cell autophagy without intact mitochondria.

## 4. Discussion

In this paper, we showed that activated autophagy increased apoptosis of T cells in SLE patients, and UC-MSCs could regulate autophagy via mitochondrial transfer. Recent studies found that aberrant autophagy played an important role in the development of autoimmune diseases [9, 19], especially in SLE. Gros et al. detected more autophagosomes in mice and human lupus T lymphocytes [12]. However, what causes aberrant autophagy in SLE patients is not clear yet. It was reported that autophagy deregulation of SLE T cells was not an immediate consequence of the inflammatory event. Autophagy of T cells from CBA/J mice could not be activated by LPS [12]. Meanwhile, another study showed that T cells from SLE patients were resistant to induction of autophagy by serum IgG from patients with SLE [11]. Consistent with these studies, we observed higher autophagy level in T cells from SLE patients than that from healthy controls, as demonstrated by LC3-IIB and p62. Additionally, T cells from SLE patients seemed less responsive to anti-CD3/CD28 antibody-induced autophagy activation. Nevertheless, we found a significant increase of LC3-IIB expression in T cells from SLE patients after anti-CD3/CD28 stimulation, following elevated respiratory mitochondrial mass. And LC3-IIB expression markedly decreased after suppression of mitochondrial accumulation. So we supposed that increased autophagy in T cells from SLE patients may be partly due to energy “starvation” [13, 20] and excessively accumulated mitochondria. Additionally, inflammatory microenvironment in SLE patients may aggravate the situation.

Furthermore, we found overactivated autophagy promoted apoptosis of T cells from SLE patients. There have been lots of studies showing the complex relationship between autophagy and apoptosis [21–26]. Generally, autophagy could rescue cells from death when they are under stress such as DNA damage [27], mitochondrial dysfunction [28], or starvation [29]. Prolonged autophagy activation leads to a high turnover rate of proteins and organelles. However, a high rate above the survival threshold may lead to cell death [14]. Previous studies suggested that peripheral T cells from SLE patients had defective mitochondria and were under energy “starvation” [13, 20]. In our study, activation of T cells induced mitochondrial biogenesis and autophagy, which seemed to increase apoptosis in contrary. However, inhibition of autophagy not always rescued T cells from apoptosis, as shown by the difference between 3-MA and chloroquine (CQ). 3-MA is a PI3K inhibitor and inhibits the initial process of autophagy, while CQ blocks lysosomal degradation, which is the late-stage of autophagy. So treatment with CQ prevented the contents in autolysosome from recycling and was unable to maintain cellular energy levels. These results further indicated the complicated relationship between autophagy and apoptosis. And additional studies must be done to elucidate the mechanism more clearly.

Interestingly, UC-MSCs could rescue T cells from SLE patients by suppressing autophagy, which was mediated by mitochondrial transfer. It has been reported before that mesenchymal stem cells (MSCs) could transfer mitochondria to tissue cells in wound healing [30]. Further studies suggested that the transfer of mitochondria was through tunneling nanotubes (TNT) from MSCs to the injured cells [31] and regulated by Miro1 [32]. It is, however, the first demonstration that they can regulate energy state of T cells from SLE patients in this way, which may reveal a novel mechanism of treating SLE patients with UC-MSCs. As we know, T cells are characteristically activated in patients with SLE, especially in those with high disease activity. Since accumulated defective mitochondria could not match the high energy consumption within T cells, they may thus induce apoptosis, subsequently increasing autoantibody formation [33]. Thus regulation of energy state of T cells could probably be a new way to treat patients with SLE.

As demonstrated, UC-MSCs showed a preference for CD4+T cells in both respiratory mitochondrial mass regulation and mitochondrial transfer. In our study, we found respiratory mitochondrial accumulation was more significant in CD4+T than in CD8+T cells after stimulation. These results indicated that mitochondrial dysfunction may play a more important role in CD4+T cell aberration. However, we need further investigations to confirm this hypothesis. Besides UC-MSCs, recent work identified platelets as potential donors of mitochondria in blood. It is found that activated platelets could release respiratory-competent mitochondria, both within membrane-encapsulated microparticles and as free organelles, which would interact with neutrophils and lead to inflammatory responses [34]. These findings suggested the relationship between mitochondria and inflammation and demonstrated the potential for treating inflammatory diseases through maintaining mitochondrial homeostasis.

Besides T cells, other cells in SLE patients, such as B cells and plasmacytoid dendritic cells [35], are also pathogenic. And it was recently reported that autophagy activation was required for plasmablast development [19]. However, whether they have defective mitochondria as T cells is not clear yet. And if UC-MSCs could regulate their autophagy in the same way still needs to be investigated.

## Figures and Tables

**Table 1:** Clinical and laboratory features in 9 patients with systemic lupus erythematosus (SLE).

Characteristics	Values
Gender (F : M)	7 : 2
Age, years	33.70 ± 4.10
Lupus duration, years	5.94 ± 2.49
SLEDAI	6.60 ± 1.89
ESR, mm/h	52.00 ± 14.90
CRP, mg/L	5.90 ± 2.95
Anti-dsDNA, %	44.44 (4/9)
Prednisone dosage, mg/day	24.78 ± 9.48
HCQ usage, %	55.56 (5/9)
ISA usage, %	33.33 (3/9)

Clinical characteristics are presented as mean ± SEM or number (%). SLEDAI, SLE disease activity index; ESR, erythrocyte sedimentation rate; CRP, C-reactive protein; HCQ, hydroxychloroquine; ISA, immunosuppressive agent. The normal ranges were as follows: ESR (0–20 mm/h) and CRP (0–8 mg/L).

## Abbreviations

AMPK: AMP-activated protein kinase
APC: Allophycocyanin
CFSE: Carboxyfluorescein succinimidyl amino ester
FITC: Fluorescein isothyocyanate
LC3: Microtubule-associated protein 1 light chain 3
LC3-IIB: Microtubule-associated protein 1 light chain 3-IIB
MDR: MitoTracker Deep Red
MG: MitoTracker Green
PBMCs: Peripheral blood mononuclear cells
PBS: Phosphate-buffered saline
PE: Phycoerythrin
SLE: Systemic lupus erythematosus
SLEDAI: SLE disease activity index
3-MA: 3-Methyladenine
ΔΨm: Mitochondrial transmembrane potential.
